# Sexual dimorphism in prokinetic effects of a ghrelin agonist acting through the lumbosacral defecation center in rats

**DOI:** 10.1186/s12576-024-00949-w

**Published:** 2024-11-22

**Authors:** Shumpei Tsukamoto, Tomoya Sawamura, Natsufu Yuki, Kazuhiro Horii, Yuuki Horii, Takeshi Homma, Shouichiro Saito, Takahiko Shiina, Yasutake Shimizu

**Affiliations:** 1 Department of Basic Veterinary Science, Laboratory of Physiology, Joint Graduate School of Veterinary Sciences, Gifu University, 1-1 Yanagido, Gifu, 501-1193 Japan; 2 Division of Biological Principles, Department of Physiology and Biophysics, Graduate School of Medicine, Gifu University, 1-1 Yanagido, Gifu, 501-1193 Japan; 3 Department of Basic Veterinary Science, Laboratory of Veterinary Anatomy, Joint Graduate School of Veterinary Sciences, Gifu University, 1-1 Yanagido, Gifu, 501-1193 Japan; 4 Division of Animal Medical Science, Center for One Medicine Innovative Translational Research (COMIT), Institute for Advanced Study, Gifu University, 1-1 Yanagido, Gifu, 501-1193 Japan

**Keywords:** Colorectal motility, Growth hormone secretagogue receptor, Preganglionic neurons, Sex differences, Spinal cord

## Abstract

We investigated the effects of a centrally penetrant ghrelin agonist, RQ-00538053, on colorectal motility in female rats in comparison with that in male rats. Intravenous administration of RQ-00538053 enhanced colorectal motility in female rats. However, approximately tenfold higher doses were required to induce responses in female rats similar to those in male rats. Higher doses were required even when the agonist was intrathecally administered to the lumbosacral spinal cord in female rats. The results of RT-qPCR showed that the level of ghrelin receptor expression in the lumbosacral spinal cord was lower in female rats than in male rats, suggesting that the lower expression level of the receptor may contribute, at least in part, to the sex differences in the action of RQ-00538053. The sexually dimorphic action of a ghrelin agonist will be important for future works aiming to utilize ghrelin agonists as novel drugs to improve constipation.

**Supplementary Information:**

The online version contains supplementary material available at 10.1186/s12576-024-00949-w.

## Background

It is widely accepted that the enteric nervous system plays a major role in the regulation of colorectal motility associated with defecation [1]. The supraspinal defecation center in the brainstem and the spinal defecation center in the lumbosacral spinal cord are also involved in the regulation of defecation [2]. We previously showed that administration of capsaicin as a noxious stimulus into the colon enhances colorectal motility by activation of the medullary raphe nuclei and the hypothalamic A11 region in male rats. The descending neurons from these nuclei to the lumbosacral spinal cord released monoamines, activating parasympathetic preganglionic neurons in male rats [3, 4]. Notably, the noxious stimulus does not enhance colorectal motility in female rats [5]. The sexually dimorphic response is due to differences in the descending neurons activated by the intracolonic noxious stimulus. In male rats, descending serotonergic and dopaminergic neurons are activated by a noxious stimulus, whereas serotonergic and GABAergic neurons are activated in female rats. GABA competes with the stimulatory action of monoamines in the spinal defecation center, so that colorectal motility is not enhanced in female rats in response to a noxious stimulus in the colon.

Several lines of evidence suggest that centrally penetrant ghrelin agonists are promising candidates of therapeutic drugs for constipation because of the potent stimulatory action on colonic motility [6–9]. The prokinetic effects of ghrelin agonists are not due to their direct action on the colon but due to activation of the spinal defecation center [8]. In support of this, it has been shown that the ghrelin receptor (growth hormone secretagogue receptor; GHSR) is expressed in parasympathetic preganglionic neurons of the lumbosacral spinal cord in male rats and that ghrelin agonists can activate these neurons [10–12]. All of the results for stimulatory effects of ghrelin agonists on colonic motility have been obtained through experiments using male rats. Considering that there is an apparent sex difference in neuronal composition operating in the spinal defecation center as mentioned above, it would be inappropriate to evaluate the potential of a ghrelin agonist as a drug to relieve constipation based on the results obtained in male rats alone.

We considered it important to verify the prokinetic effect of ghrelin agonists in female rats. For this, we investigated whether activation of GHSR by using a ghrelin agonist promotes propulsive motility of the colorectum in female rats in comparison with data obtained in male rats.

## Methods

### Animals and ethics approval

Female and male Sprague–Dawley rats (8–12 weeks of age, female rats: 200–300 g, male rats: 250–350 g) were purchased from Japan SLC (Shizuoka, Japan). The animals were housed in plastic cages at 24 ± 2 ºC with a 12-h:12-h light–dark cycle (light on at 7:00 and off at 19:00) and were allowed access to food (MF; Oriental Yeast, Tokyo, Japan) and water ad libitum. Some female rats (5 weeks old) underwent either ovariectomy (OVX) or a sham operation under mixed anesthesia with xylazine (4 mg/kg, i.m.) and ketamine (40 mg/kg, i.m.) as described previously [5]. OVX rats were used in this study to determine whether sex differences in the action of ghrelin agonist disappear with OVX, since it has been shown that sex differences in neurotransmission mechanisms in the defecation center disappeared with OVX [5]. The rats were used for experiments 3 weeks after the operation. We previously reported that descending monoaminergic neurons are activated in response to an intracolonic noxious stimulus and then colorectal motility is enhanced via the spinal defecation center in male rats, while descending GABAergic neurons are activated and consequently do not elicit enhanced colorectal motility in female rats [5]. Since the sex differences in neural composition of the spinal defecation center can be abolished by OVX, we hypothesized that the sex differences observed in the action of RQ-00538053 may also be abolished by OVX. Therefore, effects of RQ-00538053 were assessed in OVX rats.

All experimental procedures conformed to the “Regulations for Animal Experiments in Gifu University” and were approved by the president of the university after review by the Committee for Animal Research and Welfare of Gifu University (Permission No. 2021-241, 20220050, AG-P-N-20230190). The regulations of Gifu University conform to the Japanese “Act on Welfare and Management of Animals” and “Standards Relating to the Care and Keeping and Reducing Pain of Laboratory Animals (Notice of the Ministry of the Environment No. 88 of 2006)”.

### Recording of colorectal motility in vivo

Colorectal motility was recorded by an in vivo experimental system as described previously [6]. In brief, rats were anesthetized with ketamine (50 mg/kg, i.m.) and α-chloralose (60 mg/kg, into the tail vein). Anesthetics (ketamine at 1.5–2.5 mg/kg/h and 3–5 mg/kg/h and α-chloralose at 5–10 mg/kg/h and 10–20 mg/kg/h in female and male rats, respectively) were continuously infused through the femoral artery throughout the experiments. The colorectum was cannulated and filled with warm saline solution, and intraluminal pressure and expelled fluid volume were measured.

For drug administration, catheters were placed at the femoral vein, lumbar spinal cord and colorectum. A ghrelin agonist (RQ-00538053) was administered intravenously via a catheter placed in the femoral vein. In some series of experiments, RQ-00538053 was intrathecally administered into the spinal defecation center to examine the possibility that there is a sex difference in efficiency of the drug in crossing the blood–brain barrier. For intrathecal injection into the spinal defecation center, 10 µL of RQ-00538053 solution was injected with a 30 G needle connected to a polyethylene tube between the L1 and L2 vertebrae. A catheter was placed in the colorectum to administer capsaicin into the lumen of the colorectum. Capsaicin (100 nmol, approximately equal to 30 µg) was administered after dissolving in 10 µL of a solvent.

### Measurement of food intake

To investigate whether the functional property of GHSR differs between sexes in general, we focused on GHSR-mediated action other than enhancement of colorectal motility. Since the most typical action of ghrelin is enhancement of feeding [13], the effect of the agonist on food intake was assessed. Food intake was measured in the absence of prior fasting. RQ-00538053 or its solvent, saline, was administered intraperitoneally to conscious rats at 11:00, and then food intake over a 2-h period was measured. Each dose was administered to the same animal in a crossover fashion. A non-dosing period of a least 1 day was set between each treatment.

### Reverse transcription quantitative reverse transcription-PCR

The expression of GHSR mRNA in the spinal cord was assessed by reverse transcription quantitative reverse transcription PCR (RT-qPCR) as reported previously [14]. Briefly, the T13-S2 spinal cord was collected and frozen in liquid nitrogen before storage at − 80 °C. Frozen tissue was homogenized, and total cellular RNA was extracted with TRI reagent (Molecular Research Center, Inc., Cincinnati, OH). First-strand cDNA was synthesized from 3 µg of total RNA with SuperScript III reverse transcriptase and Random primers (Thermo Fisher Scientific). RT-qPCR was performed using the Applied Biosystems StepOnePlus Real-Time PCR System (Thermo Fisher Scientific) and Power SYBR Green Master Mix (Thermo Fisher Scientific). The expression of β-actin served as an internal control. The following primers specific for GHSR cDNA were used: 5′-CCAGAACCACAAGCAGACAGTG-3′ (forward) and 5′-GAAGAGGACAAAGGACACCAGG-3′ (reverse). Two variants of GHSR, GHSR-1a and GHSR-1b, are present in humans [15]. Although it is unclear whether GHSR-1b is expressed in rats as we cannot find the variant in the database, the probes used were designed to specifically amplify GHSR-1a based on the human sequence. The primers specific for β-actin cDNA were 5′-CATCCGTAAAGACCTCTATGCCAAC-3′ (forward) and 5′-ATGGAGCCACCGATCCACA-3′ (reverse).

### In situ hybridization (ISH) and immunofluorescent histological analysis

After rats had been anesthetized by urethane and euthanized, they were perfused transcardially with Ringer solution containing 0.1% heparin followed by 4% paraformaldehyde (PFA) in 0.1 M phosphate-buffered saline (PBS, pH 7.4). The spinal cord of each rat was collected and immersed in the same fixative overnight. Then the spinal cord was soaked in 30% sucrose in 0.1 M phosphate buffer (PB, pH 7.4), frozen with O.C.T. compound medium (Sakura Finetech, Tokyo, Japan), sectioned at 20 µm in thickness with a cryostat (CM 1850; Leica Biosystems, Wetzlar, Germany), and stored at − 25 ºC until use.

ISH was performed as reported previously [16]. Briefly, the forward (5′-GCAACCTGCTCACTATGCTG-3′) and reverse (5′-GTCTCCGCCATAGCTTCCTC-3′) primers of rat GHSR (NM_032075.3) for ISH were determined by preliminary experiments, and the forward primer with the T7 promoter sequence (5′-TAATACGACTCACTATAGGG-3′) and the reverse primer with the Sp6 promoter sequence (5′-ATTTAGGTGACACTATAGAA-3′) in their 5′ terminal regions were made commercially (Fasmac Co., Ltd., Kanagawa, Japan). Using these primers and rat cDNA prepared as described above, PCR was performed and the purified PCR product was obtained. Then digoxigenin (DIG)-labeled sense and antisense RNA probes were synthesized from the purified PCR product using a DIG RNA Labeling Kit (Roche Diagnostics GmbH, Mannheim, Germany), and the synthesized RNA transcripts were aliquoted into 100 ng/µl by RNase-free water and stored at -60ºC until use. Cryostat sections prepared as described above were dried and fixed again with 4% PFA in 0.1 M PBS for 20 min, washed in PBS, and incubated with 20 µg/ml of proteinase K (Dako North America Inc., CA, USA) in PBS at 37 ºC for 30 min. After rinsing in cold PBS, the sections were acetylated in RNase-free water containing 1.35% triethanolamine, 0.25% acetic anhydrite, and 0.058% HCl for 10 min. After rinsing in PBS, the sections were dehydrated by an ascending graded ethanol series and dried, then applied a DIG-labeled RNA probe diluted into 1.0 µg/mL by hybridization buffer (50% formamide, 20% dextran sulfate, 2% blocking reagent (Roche), 0.01% N-lauroylsarcosine (NLS), and 0.01% sodium lauryl sulfate in 5 × saline sodium citrate buffer (SSC, pH 7.4), and hybridized in an incubator at 55ºC overnight. After hybridization, the sections were washed in 2 × SSC containing 50% formamide and 0.01% NLS at 55 ºC for 30 min each and incubated with 20 µg/ml of RNase A (Roche) in NTE buffer (500 mM NaCl, 10 mM trimethylolaminomethane hydrochloride (Tris), and 1 mM ethylenediaminetetraacetic acid, pH 8.0) at 37 ºC for 30 min. After rinsing in 2 × SSC containing 0.01% NLS at 37 ºC for 30 min twice and 25 mM Tris-buffered saline (TBS, pH 7.4) containing 0.05% Tween 20, the sections were incubated with 2% normal sheep serum (Sigma-Aldrich Co., MO, USA) and 1% blocking reagent (Roche) in TBS at room temperature (RT) for 1 h, followed by incubation with an alkaline phosphatase-conjugated sheep anti-DIG antibody (Roche) diluted 1:2000 at 4 ºC overnight. After rinsing in TBS, the sections were colorized by 2% NBT/BCIP (Nitro blue tetrazolium chloride/5-bromo-4-chloro-3-indolyl phosphate toluidine salt) stock solution (Roche) in colorization buffer (0.1 M Tris, 0.1 M NaCl, 50 mM MgCl_2_ and 0.2% Tween 20, pH 9.5) at 20 ºC overnight and then dehydrated, cover-slipped, and observed under a differential interference contrast microscope (Nikon Eclipse E600, Tokyo, Japan). Since the probes for ISH were designed with the common sequence portion of human GHSR-1a and GHSR-1b, both of them would be stained if there are two types of GHSRs in rats.

Some hybridized sections were further processed for immunofluorescence using goat anti-Choline Acetyltransferase (ChAT) antibody (AB144P, Sigma-Aldrich). After colorization in ISH, the sections were incubated with 2% normal donkey serum at RT for 30 min, rinsed in PBS, and incubated with anti-ChAT antibody diluted 1:150 at 4 ºC overnight. After rinsing in PBS, the sections were incubated with Alexa Fluor 594-conjugated donkey anti-goat IgG (Jackson ImmunoResearch Laboratories, West Grove, PA, USA) diluted 1:100 at RT for 90 min. The sections were then counterstained with DAPI (Dojindo Laboratories, Kumamoto, Japan) diluted 1:5000 at RT for 10 min, cover-slipped, and observed under a fluorescence microscope (Z-X810; Keyence, Osaka, Japan). A digital image of the hybridization signal in the section was taken by Z-X810 under the phase-contrast bright field, and the hybridization signal was converted into green and overlapped with red immunofluorescence by Adobe Photoshop Elements 2020 (Adobe Systems Inc., San Jose, CA, USA). Negative controls were created by replacing the primary antibody with normal rabbit IgG (Fujifilm Wako Pure Chemical Co.) and no specific staining was observed in the control sections.

### Reagents

RQ-00538053 supplied by RaQualia Pharma Inc. was used as a ghrelin agonist. Capsaicin (Sigma-Aldrich), ketamine (Daiichi Sankyo, Tokyo, Japan), α-chloralose (Nacalai Tesque, Kyoto, Japan), and xylazine (Bayer Yakuhin, Osaka, Japan) were also used. RQ-00538053 was dissolved in saline for administration. α-Chloralose was dissolved in 2-hydroxypropyl-β-cyclodextrin (FUJIFILM Wako Pure Chemical, Osaka, Japan) and then made up with saline for infusion. Capsaicin was dissolved in ethanol and then made up with saline containing 10% Tween 80 for administration.

### Data analysis

Colorectal motility induced by intravenous or intrathecal administration of RQ-00538053 was assessed by using data for 10 min from the first contractile response with expulsion of intraluminal fluid after the administration. The number of contractions with expulsion of intraluminal fluid was counted. Data are presented as means ± SD. Statistical analysis of two groups was performed by the unpaired Student’s t-test. Statistical analysis of multiple groups was performed by the Tukey test. P-values less than 0.05 were considered statistically significant differences.

## Results

### Effects of intravenous administration of RQ-00538053 on colorectal motility in female and male rats

**Fig. 1:**
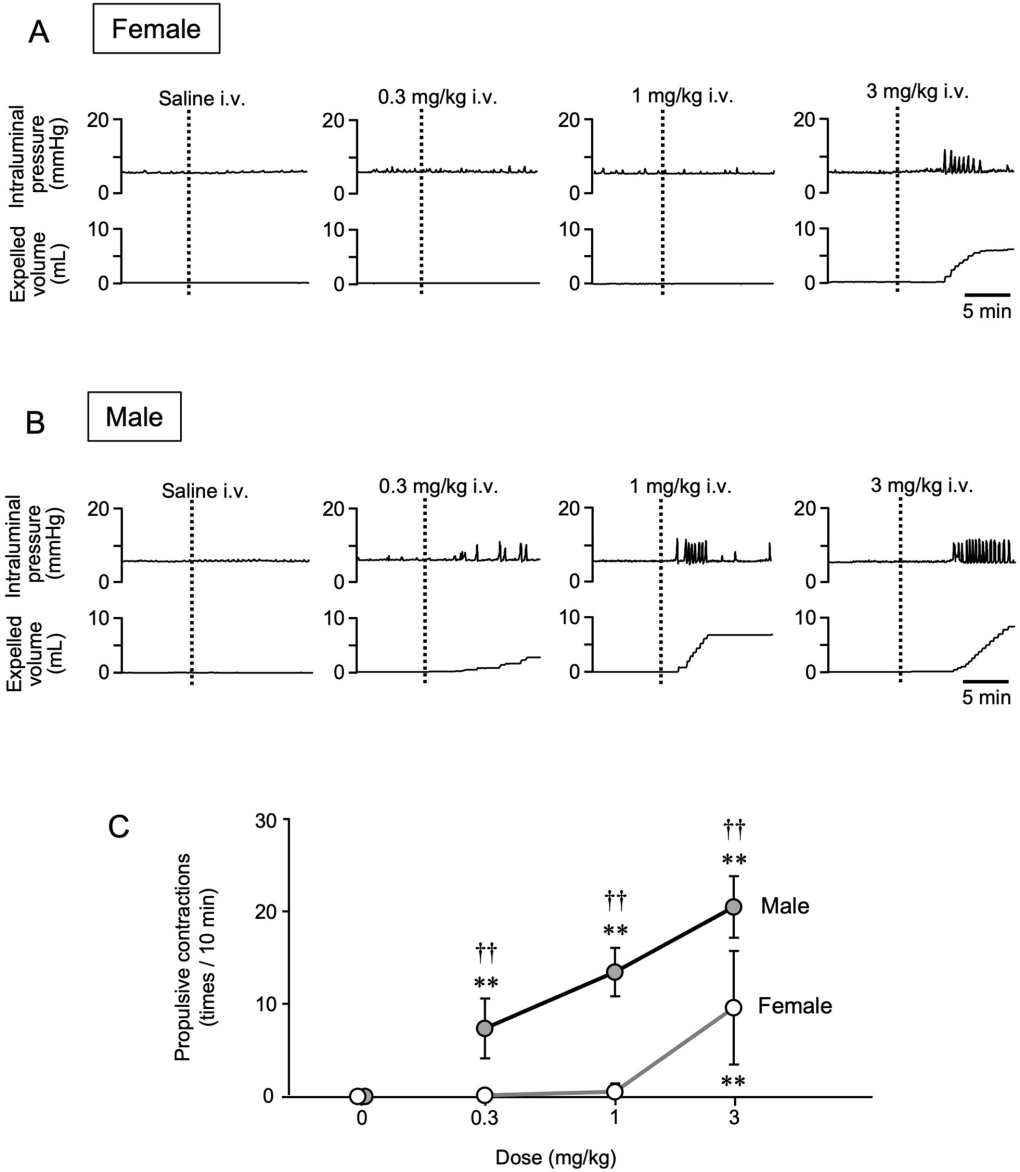
Effects of intravenous administration of RQ-00538053 on colorectal motility in male and female rats. Representative recording traces of colorectal pressure (upper panel) and expelled volume (lower panel) before and after intravenous (i.v.) administration of RQ-00538053 at the doses of 0 (vehicle control), 0.3, 1 and 3 mg/kg in female (**A**) and male (**B**) rats are shown. Summarized graphs of the number of propulsive contractions (contractions with expulsion of saline/10 min) are shown (**C**). Each value represents the mean ± SD (n = 5). **P < 0.01, significantly different from each vehicle control. ^††^P < 0.01 significantly different between female and male rats

To see whether activation of GHSR in the spinal cord promotes propulsive motility of the colorectum, a ghrelin agonist, RQ-00538053, was intravenously administered at three doses (0.3, 1 and 3 mg/kg) in anesthetized rats. Intravenous administration of a vehicle solution had no effect on colorectal motility in both female and male rats. In female rats, colorectal motility was enhanced by RQ-00538053 at a dose of 3 mg/kg but not at doses of 0.3 and 1 mg/kg (Fig. 1A). The numbers of propulsive contractions at the doses of 0 (vehicle control), 0.3, 1 and 3 mg/kg were 0.0 ± 0.0, 0.0 ± 0.0, 0.4 ± 0.9 and 9.4 ± 6.1, respectively (Fig. 1C, n = 5). In male rats, on the other hand, an apparent enhancement of colorectal motility was observed when RQ-00538053 was administered intravenously at a dose as low as 0.3 mg/kg, and increasing the dose elicited greater responses in a dose-dependent manner (Fig. 1B). The numbers of propulsive contractions at the doses of 0 (vehicle control), 0.3, 1 and 3 mg/kg were 0.0 ± 0.0, 7.2 ± 3.2, 13.2 ± 2.6 and 20.2 ± 3.3, respectively (Fig. 1C, n = 5). There was a significant difference in the number of contractions after RQ-00538053 administration between female and male rats at all doses (P = 0.0091 at the dose of 0.3 mg/kg, P < 0.0001 at the dose of 1 mg/kg, and P < 0.0001 at the dose of 3 mg/kg, n = 5).

### Effects of intrathecal administration of RQ-00538053 on colorectal motility in female and male rats

**Fig. 2:**
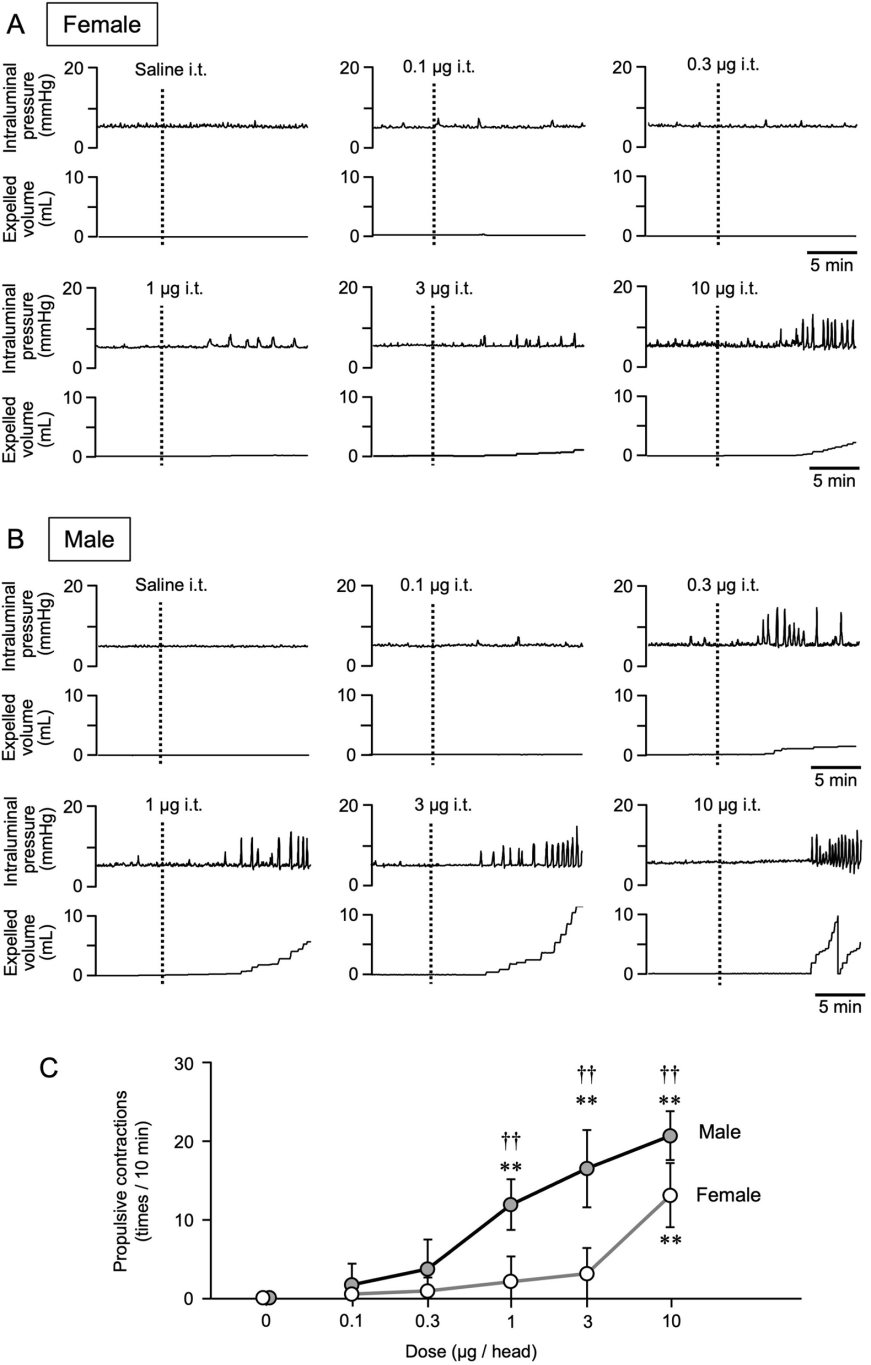
Effects of intrathecal administration of RQ-00538053 on colorectal motility in male and female rats. Representative recording traces of colorectal pressure (upper panel) and expelled volume (lower panel) before and after intrathecal (i.t.) administration of RQ-00538053 at the doses of 0 (vehicle control), 0.1, 0.3, 1, 3 and 10 µg in female (**A**) and male (**B**) rats are shown. Summarized graphs of the number of propulsive contractions (contractions with expulsion of saline/10 min) are shown (**C**). Each value represents the mean ± SD (n = 5). **P < 0.01, significantly different from each vehicle control. ^††^P < 0.01 significantly different between female and male rats

We examined the possibility that the less potent action of RQ-00538053 in female rats is due to low efficiency of the drug in crossing the blood–brain barrier. RQ-00538053 was administered intrathecally at the spinal cord segment L6-S1 at 5 doses (0.1, 0.3, 1, 3 and 10 µg) in anesthetized rats and colorectal motility was evaluated. Intrathecal administration of a vehicle solution had no effect on colorectal motility in female and male rats. Administration of RQ-00538053 elicited an increase in colorectal motility in a dose-dependent manner in male rats (Fig. 2B). The numbers of propulsive contractions at the doses of 0 (vehicle control), 0.1, 0.3, 1, 3 and 10 µg were 0.0 ± 0.0, 1.8 ± 2.7, 3.8 ± 3.8, 12.0 ± 3.2, 16.6 ± 4.9, and 20.8 ± 3.1, respectively (Fig. 2C, n = 5). In female rats, a concentration-dependent increase in colorectal motility was also observed (Fig. 2A), but approximately tenfold higher doses were required to obtain effects comparable to those in male rats. The numbers of propulsive contractions at the doses of 0 (vehicle control), 0.1, 0.3, 1, 3 and 10 µg were 0.0 ± 0.0, 0.6 ± 1.3, 1.0 ± 1.7, 2.2 ± 3.2, 3.2 ± 3.3 and 13.2 ± 4.1, respectively (Fig. 2C, n = 5). There was a significant difference in the number of contractions between female and male rats at the dose of 1 µg or higher (P = 0.0002 at the dose of 1 µg, P < 0.0001 at the dose of 3 µg, and P = 0.0083 at the dose of 10 µg, n = 5).

### Effect of OVX on sexually dimorphic response of colorectal motility to RQ-00538053

**Fig. 3:**
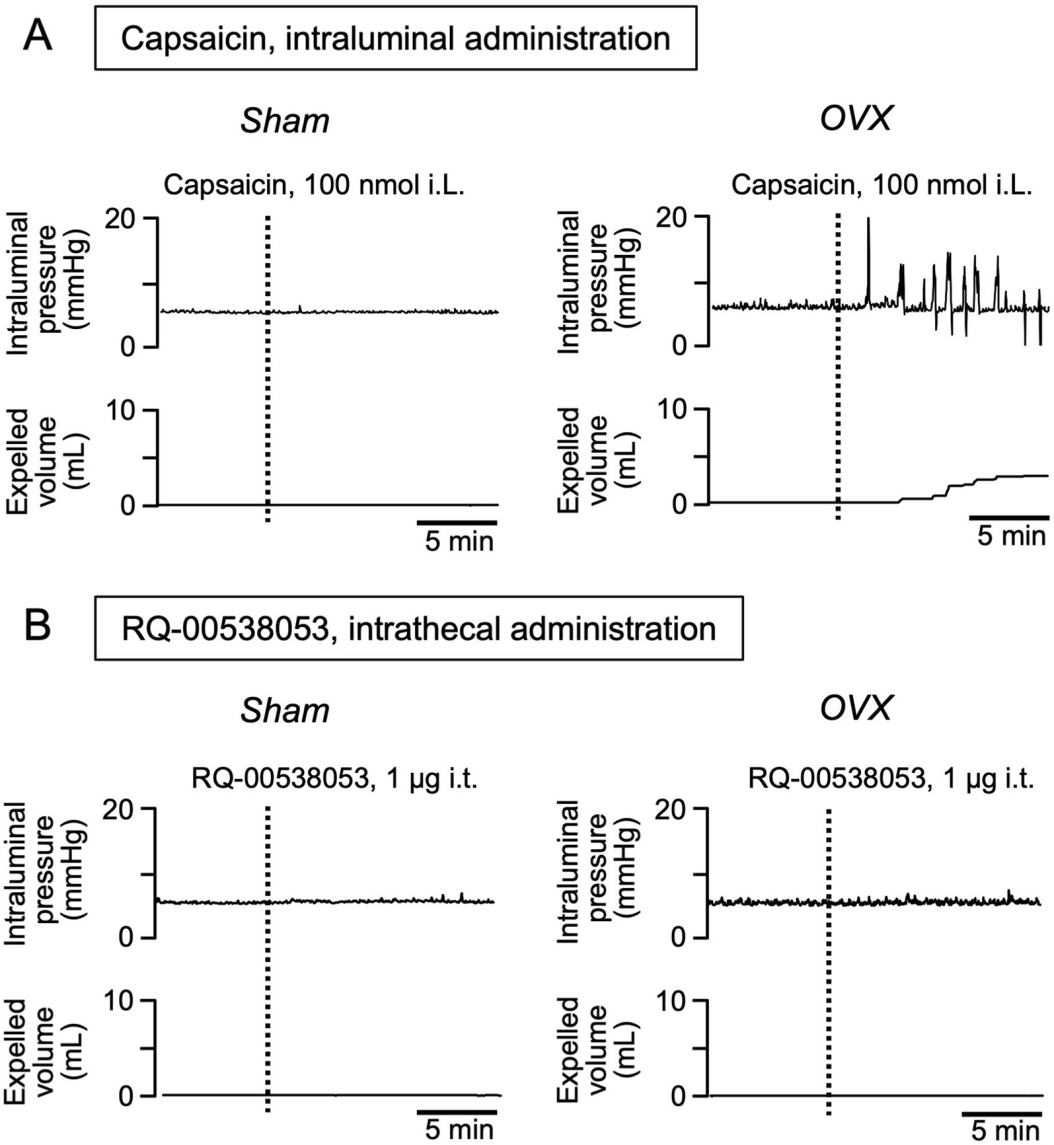
Effect of intrathecal administration of RQ-00538053 on colorectal motility after OVX in rats. Representative recording traces of colorectal pressure (upper panel) and expelled volume (lower panel) before and after intrathecal (i.t.) administration of 1 µg RQ-00538053 (**A**) or intraluminal (i.L.) administration of 100 nmol capsaicin (**B**) in sham and OVX rats are shown. Similar results were reproducibly obtained in five independent rats in each experiment

The effects of RQ-00538053 were assessed in OVX rats to see whether sex differences in the action of the ghrelin agonist disappear with OVX. In this experiment, we used the maximal concentration at which there was no clear response in intact females (i.e., 1 µg, see Fig. 2A) and we expected that a response would be elicited if the putative inhibitory mechanism was eliminated by OVX. In sham-operated control female rats, capsaicin administration into the colon did not enhance colorectal motility, while the motility was enhanced by capsaicin 3 weeks after OVX (Fig. 3A). These results indicate that a period of 3 weeks after OVX is sufficient to convert operative descending neurons from the female phenotype to the male phenotype. Nevertheless, intrathecal administration of RQ-00538053 failed to enhance colorectal motility in OVX rats as in sham rats (Fig. 3B). Similar results were reproducibly obtained in five independent rats in each experiment.

### Effects of RQ-00538053 on food intake in female and male rats

**Fig. 4:**
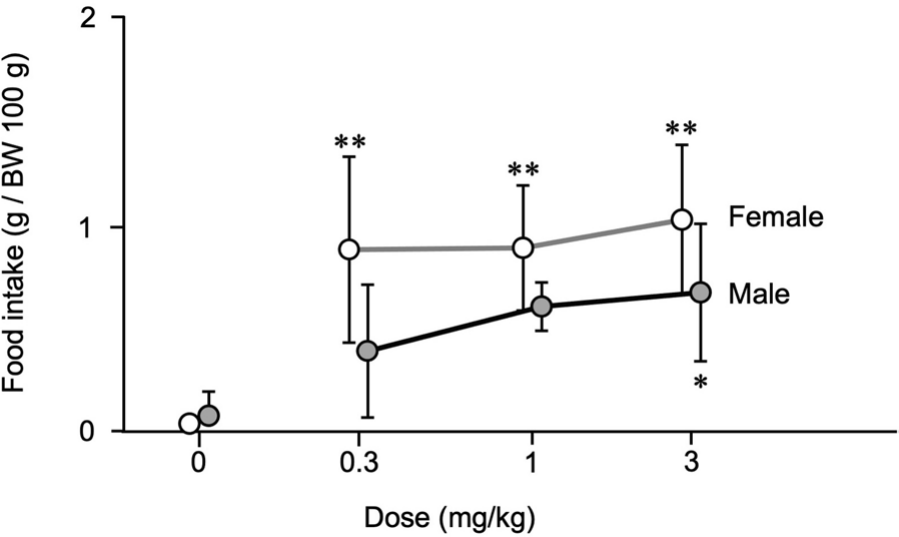
Effects of intraperitoneal administration of RQ-00538053 on food intake in male and female rats. Summarized graphs of relative food intake (g per 100 g body weight) 2 h after intraperitoneal administration of RQ-00538053 at the doses of 0 (vehicle control), 0.3, 1 and 3 mg/kg in female and male rats. Each value represents the mean ± SD (n = 5). *P < 0.05, **P < 0.01, significantly different from each vehicle control. There was no significant difference between female and male rats

We considered the possibility that the sex differences in the action of RQ-00538053 on colorectal motility are due to sex differences in the functional property of GHSR. To investigate this possibility, we examined whether there are sex differences in other GHSR-mediated responses. Since the most typical action of ghrelin is enhancement of feeding [13], the effect of the agonist on food intake was assessed. Food intake was evaluated in conscious female and male rats for 2 h after intraperitoneal administration of RQ-00538053 at 3 doses (0.3, 1 and 3 mg/kg). Relative amounts of food intake (expressed as g per 100 g body weight) 2 h after intraperitoneal administration of RQ-00538053 at the doses of 0 (vehicle control), 0.3, 1 and 3 mg/kg were 0.01 ± 0.02, 0.87 ± 0.46, 0.88 ± 0.31 and 1.02 ± 0.37, respectively, in female rats and 0.05 ± 0.12, 0.37 ± 0.33, 0.59 ± 0.12 and 0.66 ± 0.34, respectively, in male rats (Fig. 4, n = 5). In female rats, food intake was significantly increased from the lowest dose (0.3 mg/kg, P = 0.0007) compared with that in female rats administered the vehicle saline, while food intake was significantly increased only at the highest dose (3 mg/kg, P = 0.0276) in male rats (Fig. 4).

### Expression of GHSRs in the lumbosacral spinal cord in female and male rats

**Fig. 5:**
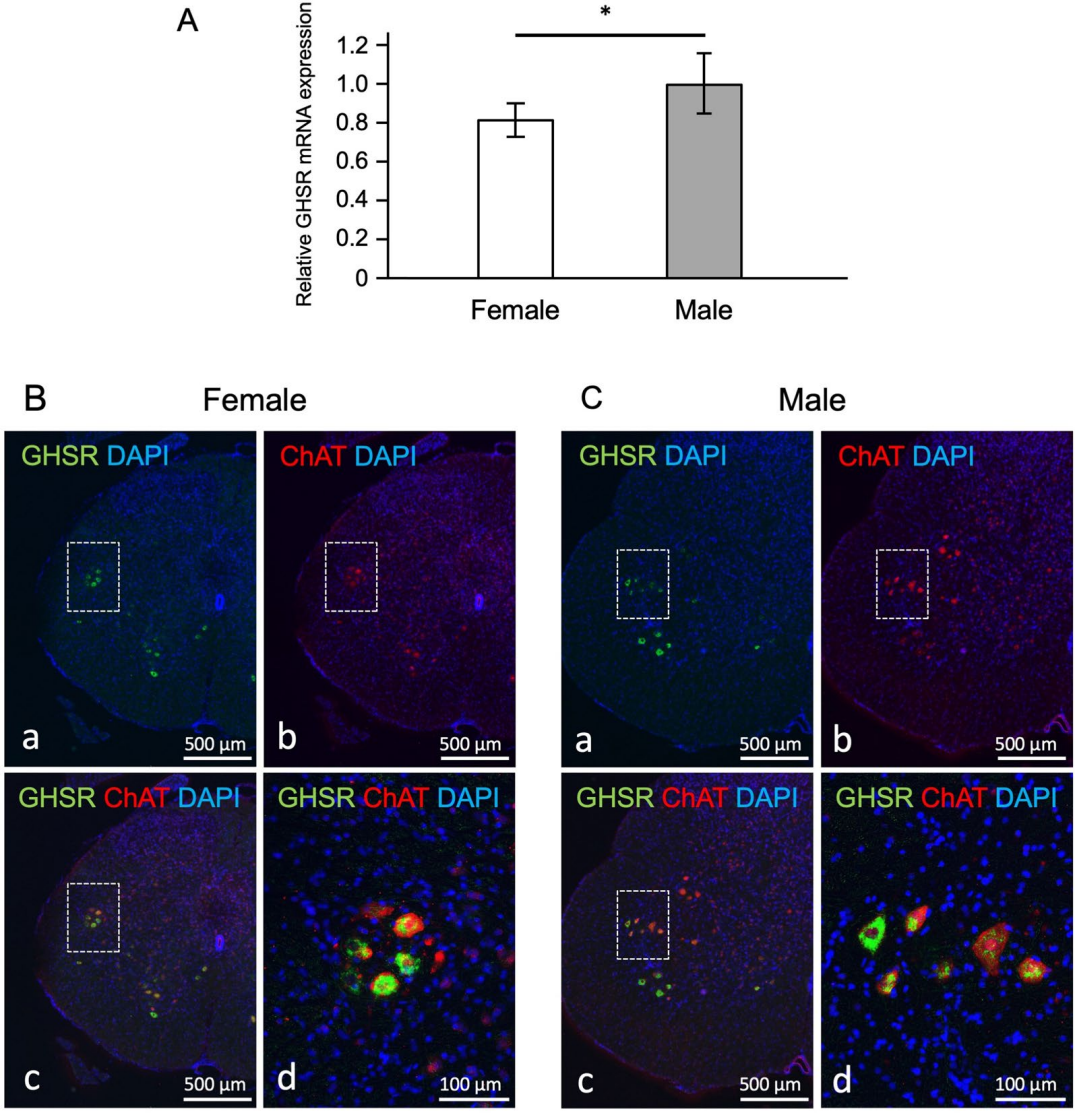
Expression of GHSRs in the lumbosacral spinal cord in female and male rats. Results of quantitative PCR analysis of GHSR mRNA expression in the lumbosacral spinal cord are shown (**A**). β-actin was used as an internal control. The ratio of GHSR to β-actin was determined and summarized as bar graphs (n = 6). The error bars indicate SD. *P < 0.05, significant difference between female and male rats. Immunofluorescence for choline acetyltransferase (ChAT) after in situ hybridization for GHSR mRNA in the lumbosacral spinal cord in female (**B**) and male (**C**) rats are shown. The nuclei of all neurons were counterstained with DAPI (blue). **a** The expression of GHSR mRNA in the spinal cord shown in green-color. **b** ChAT immunofluorescence in the spinal cord shown in red-color. **c** The merged image of GHSR mRNA expression and ChAT immunofluorescence. Boxes in (**a**)–(**c**) represent the intermediolateral cell columns (IML). **d** High magnification of boxed area in (**c**)

To determine whether the less potent action of RQ-00538053 in female rats is due to low expression of GHSR in the lumbosacral spinal cord, we performed RT-qPCR experiments. The probes used were designed to specifically amplify GHSR-1a based on the human sequence. The transcriptional level of GHSR in the lumbosacral spinal cord of female rats was significantly lower than that in male rats (Fig. 5A; relative values for female and male rats: 0.82 ± 0.09 and 1.00 ± 0.16, respectively, P = 0.0132, n = 6).

Since it has already been shown that GHSR is expressed predominantly in preganglionic neurons originating from the spinal cord [10, 11], the results obtained by RT-qPCR may reflect expression levels in preganglionic neurons. In the present study, we also verified whether expression of GHSR was mainly in preganglionic neurons by ISH. The hybridization signal for GHSR mRNA was observed in the intermediolateral cell columns (IML; boxes in Fig. 5B-a and C-a also see Supplementary Fig. 1), around the central canal and in the ventral horns in the spinal cord of both female rats (Fig. 5B) and male rats (Fig. 5C). ChAT immunoreactivity, as a marker of preganglionic neurons, was observed widely in the spinal cord, and neurons in IML showed intense ChAT immunoreactivity in both sexes (boxes in Fig. 5B-b in female rats and 5C-b in male rats). In the IML, 85% and 90% of the GHSR mRNA-expressing cells showed ChAT immunoreactivity in female and male rats, respectively (Fig. 5B-c and B-d in female rats; 5C-c and C-d in male rats).

## Discussion

We previously showed that there is an apparent sex difference in neural composition of the spinal defecation center and that the motility response of the colorectum to a noxious stimulus into the colon substantially differs in female and male rats [5]. Although ghrelin agonists are expected to be potential drugs for improving constipation, prokinetic actions of the agonists have only been proven only in male animals. In the present study, we investigated whether a ghrelin agonist promotes propulsive motility of the colorectum in female rats. One of the key findings is that intravenous administration of a centrally penetrant ghrelin agonist, RQ-00538053, enhances colorectal motility in female rats. Remarkably, approximately tenfold higher doses of RQ-00538053 were required in female rats to observe the same level of increased colorectal motility as that in male rats. Although the prokinetic action of the agonist was less potent in female rats, the effective dose for promoting colonic motility (Fig. 1; 3 mg/kg) is comparable to the doses reported in previous studies using other ghrelin agonists in male rats (*e.g.*, CP464709, 5 mg/kg i.v., [6]; capromorelin, 4 mg/kg i.v., [17]; ulimorelin, 5 mg/kg i.v., [8]; HM01, 1 mg/kg i.v., [18]). Accordingly, it seems reasonable to consider that centrally penetrant ghrelin agonists have potential as new drugs for alleviating constipation also in females.

Sex differences are known to exist in the function of the blood–brain barrier [19]. Since intravenously administered RQ-00538053 needs to pass through the blood–brain barrier to exert its action, the process by which the drug reaches the receptor site may be a possible determinant of the potency of its action. We found that even when the agonist was intrathecally administered to the lumbosacral spinal cord, higher doses were required in female rats to enhance colorectal motility as was observed with intravenous administration. These results suggest that the sexually dimorphic response of colorectal motility is not dependent on the efficiency of the agonist crossing the blood–brain barrier.

We previously reported that the composition of descending neurons operating in response to an intracolonic noxious stimulus differs between sexes, while the sex difference is eliminated by OVX [5]. These findings suggest essential roles of female sex hormones in generation of the sex difference. In the present study, female rats 3 weeks after OVX were used to examine the effects of female hormones. Since intracolonic capsaicin administration elicited a colorectal motility response in the OVX rats, though intact female rats did not show enhanced motility (Fig. 3A), it is rational to consider that 3 weeks is a sufficient time for the neural composition in the spinal defecation center to change from female type (*i.e.*, descending GABAergic neurons being active and thus colorectal motility not being enhanced in response to capsaicin) to male type (*i.e.*, descending monoaminergic neurons being mainly operative and thus colorectal motility being enhanced in response to capsaicin) [5]. In contrast, the sex differences in the action of RQ-00538053 remained unchanged 3 weeks after OVX (Fig. 3B). It seems thus likely that the less potent action of RQ-00538053 in female rats is unrelated to the female type of neural composition in the spinal defecation center. Taken together, it is probable that the sexually dimorphic action of RQ-00538053 is not associated with the action of female sex hormones.

It is possible that the higher dose of RQ-00538053 required to elicit an apparent reaction of the colorectum in female rats is due to sex differences in functional property of GHSR (*e.g.*, GHSR in female rats being less sensitive to its ligands). If this is the case, it is expected that similar sex differences can be observed in other GHSR-mediated actions. To investigate this possibility, the effect of RQ-00538053 on food intake was assessed because enhancement of feeding is the most typical action of ghrelin [13]. Food intake was measured from 11:00 to 13:00 under non-fasting conditions to observe agonist-induced feeding preferentially. The results obtained showed that the effects of RQ-00538053 in promoting feeding were equivalent or rather greater in female rats (Fig. 4), excluding the possibility that the receptor function in female rats is generally less potent.

The results of RT-qPCR showed that the level of GHSR expression in the lumbosacral spinal cord was lower in female rats than in male rats. It has been demonstrated that GHSR is expressed predominantly in preganglionic neurons originating from the spinal cord [10, 11]. Since our ISH experiments provided similar results, it is appropriate to consider that the results obtained by RT-qPCR may reflect expression levels in preganglionic neurons. Hence, the lower expression level of GHSR in female rats may contribute, at least in part, to the sex differences in the action of RQ-00538053. It has been reported that there are sex differences in expression levels of several G protein-coupled receptors [20]. For example, it was shown that the protein levels of μ opioid receptors in the spinal cord were significantly higher in male rats than in female rats, whereas significantly higher concentrations of κ opioid receptors were found in female rats [21]. Our findings provide an additional example of sex-dependent expression level of receptors.

## Conclusion

Our findings revealed that a centrally penetrant ghrelin agonist, RQ-00538053, enhances colorectal motility in female rats. Notably, the findings that higher doses of a ghrelin agonist are required in female rats to elicit the same levels of colonic motility as those in male rats are important because these findings suggest that there may be differences in the effective doses for men and women when ghrelin agonists are used for treatment of constipation. Overall, the data obtained in this study will provide important information for future works aiming to utilize ghrelin agonists as novel drugs to improve constipation.

## Supplementary Information

Additional file 1.

## Data Availability

The datasets used and/or analyzed during the current study are available from the corresponding author on reasonable request.

## Abbreviations

GHSR: Growth hormone secretagogue receptor
OVX: Ovariectomy
RT-qPCR: Reverse transcription quantitative reverse transcription PCR
ISH: In situ Hybridization
PFA: Paraformaldehyde
PBS: Phosphate-buffered saline
PB: Phosphate buffer
DIG: Digoxigenin
NLS: N-lauroylsarcosine
SSC: Saline sodium citrate buffer
TBS: Tris-buffered saline
RT: Room temperature
NBT/BCIP: Nitro blue tetrazolium chloride/5-bromo-4-chloro-3-indolyl phosphate toluidine salt
ChAT: Choline acetyltransferase
DAPI: 4',6-Diamidino-2-phenylindole
IML: Intermediolateral cell columns
