# Supplementary material for: Sexual dimorphism in prokinetic effects of a ghrelin agonist acting through the lumbosacral defecation center in rats
Source: J Physiol Sci. 2024 Nov 22;74:54. doi: 10.1186/s12576-024-00949-w (PMC11583643; doi:10.1186/s12576-024-00949-w)
Supplement: Supplementary file 1 — Additional file 1. [file 12576_2024_949_MOESM1_ESM.pdf]

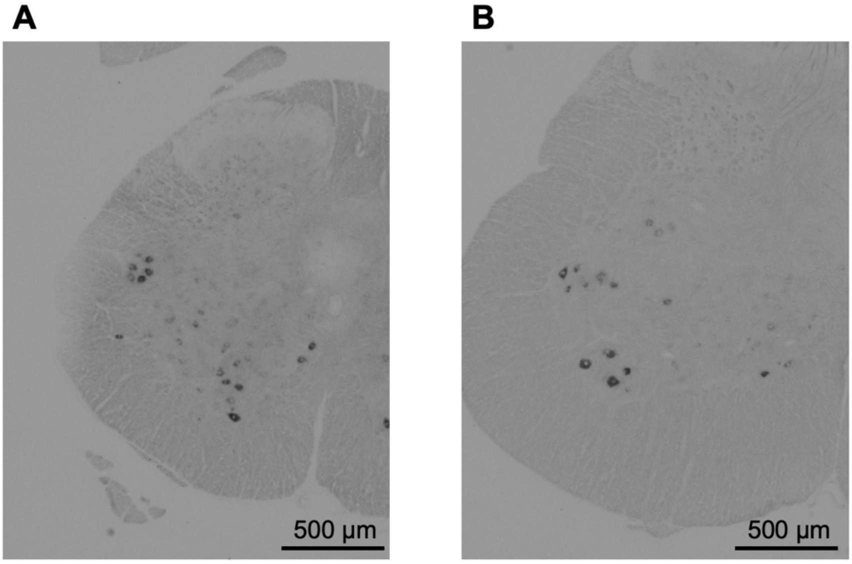

Supplementary Figure 1. Detection of GHSRs by ISH in the lumbosacral spinal cord in female and male rats

Results of *in situ* hybridization for growth hormone secretagogue receptor (GHSR) mRNA in the lumbosacral spinal cord in female (A) and male (B) rats are shown. Digoxigenin (DIG)-labeled RNA probes were used. The hybridized sections were incubated with an alkaline phosphatase-conjugated sheep anti-DIG antibody and were colorized by NBT/BCIP. A digital image of the hybridization signal in the section was taken by Z-X810 under the phase-contrast bright field, and the hybridization signal was converted into green. The converted green image is shown in Fig. 5.
